# Transcriptome profiling reveals Th2 bias and identifies endogenous itch mediators in poison ivy contact dermatitis

**DOI:** 10.1172/jci.insight.124497

**Published:** 2019-07-25

**Authors:** Boyi Liu, Yan Tai, Boyu Liu, Ana I. Caceres, Chengyu Yin, Sven-Eric Jordt

**Affiliations:** 1 Department of Neurobiology and Acupuncture Research, The Third Clinical Medical College, Zhejiang Chinese Medical University, Key Laboratory of Acupuncture and Neurology of Zhejiang Province, Hangzhou, China.; 2 Academy of Chinese Medical Sciences, Zhejiang Chinese Medical University, Hangzhou, China.; 3 Department of Anesthesiology, Duke University School of Medicine, Durham, North Carolina, USA.

**Keywords:** Immunology, Neuroscience, Cytokines, Pharmacology

## Abstract

In the United States, poison ivy is the most common naturally occurring allergen that causes allergic contact dermatitis (ACD). The immune and pruritic mechanisms associated with poison ivy ACD remain largely unexplored. Here, we compared skin whole transcriptomes and itch mediator levels in mouse ACD models induced by the poison ivy allergen, urushiol, and the synthetic allergen, oxazolone. The urushiol model produced a Th2-biased immune response and scratching behavior, resembling findings in poison ivy ACD patients. Urushiol-challenged skin contained elevated levels of the cytokine thymic stromal lymphopoietin (TSLP), a T cell regulator and itch mediator, and pruritogenic serotonin (5-HT) and endothelin (ET-1) but not substance P (SP) or histamine. The oxazolone model generated a mixed Th1/Th2 response associated with increased levels of SP, 5-HT, and ET-1 but not TSLP or histamine. Injections of a TSLP monoclonal neutralizing antibody or serotonergic or endothelin inhibitors, but not SP inhibitors or antihistamines, reduced scratching behaviors in urushiol-challenged mice. Our findings suggest that the mouse urushiol model may serve as a translational model of human poison ivy ACD. Inhibiting signaling by TSLP and other cytokines may represent alternatives to the standard steroid/antihistamine regimen for steroid-resistant or -intolerant patients and in exaggerated systemic responses to poison ivy.

## Abstract 2

Characterization of the immune and pruritic pathways in a mouse model of poison ivy-induced allergic contact dermatitis.

## Introduction

Allergic contact dermatitis (ACD) is a common skin condition resulting from cutaneous contact with an allergen (1). In the United States, the most common naturally occurring allergen-induced ACD is caused by contact with poison ivy and related plants, including poison sumac and poison oak (2, 3). The major allergen in these plants is urushiol, concentrated in the oleoresinous sap (4). It is estimated that more than 10 million Americans suffer from poison ivy ACD every year (3, 5). Poison ivy ACD also accounts for 10% of all lost-time injuries in the US Forest Service (3, 5). Elevated atmospheric CO_2_ levels and climate change have increased the biomass and geographic range of poison ivy and urushiol content, resulting in more toxic plants (6). These effects will likely increase the incidence of poison ivy ACD in the future (7).

Clinical manifestations of poison ivy–induced ACD include intense and persistent itch (pruritus), burning and pain sensation, rashes, and swelling, followed by vesicles in severe cases (8). Skin inflammation and pruritus usually last for weeks. The intense pruritus often forces scratching behavior that injures the skin, exacerbates inflammation, and may lead to infections (9). Patients are usually treated with steroids and antihistamines. However, antihistamines are mostly ineffective for treating itch resulting from poison ivy ACD, and some patient populations poorly tolerate extended high-level steroid treatment (10).

The mechanisms underlying pruritus and skin inflammation of poison ivy ACD remain poorly understood because of limited clinical data and animal studies. Most of the mechanistic understanding of ACD is based upon animal models using experimental allergens not present in the environment, such as oxazolone, dinitrofluorobenzene (DNFB), or squaric acid dibutylester (SADBE) (11–13). Recently, it has become evident that different allergens elicit widely divergent immune responses in both humans and animals, suggesting these models may not be representative of environmental forms of ACD (14, 15). Given the high incidence and public health impact of poison ivy–induced ACD, it is necessary to establish and characterize a clinically relevant animal model using the actual allergen urushiol.

In a previous study, we successfully established a mouse model of poison ivy ACD using the allergen urushiol (16). This mouse model recapitulates key hallmarks of human poison ivy ACD, including skin edema, inflammation, and itch responses. Recently, we identified a crucial role for the cytokine IL-33 in mediating itch responses in the urushiol mouse model, through its receptor, ST2, that we detected in peripheral sensory neurons known to mediate itch sensations (17, 18). However, blocking IL-33/ST2 did not completely eliminate itch-related behavior, suggesting other unknown itch pathways remain active in this model (17).

Here, we aimed to further characterize the cutaneous immune response in the urushiol ACD model by using transcriptome microarray, quantitative PCR (qPCR) expression profiling, and skin pathology evaluation. We compared the urushiol model with the widely used oxazolone-induced ACD model. We further explored the itch mechanisms active in the urushiol model by pharmacological intervention and behavioral analysis, aiming to identify antipruritic strategies for poison ivy ACD.

## Results

### Establishment and comparison of oxazolone- and urushiol-induced ACD in mice.

**Figure 1:**
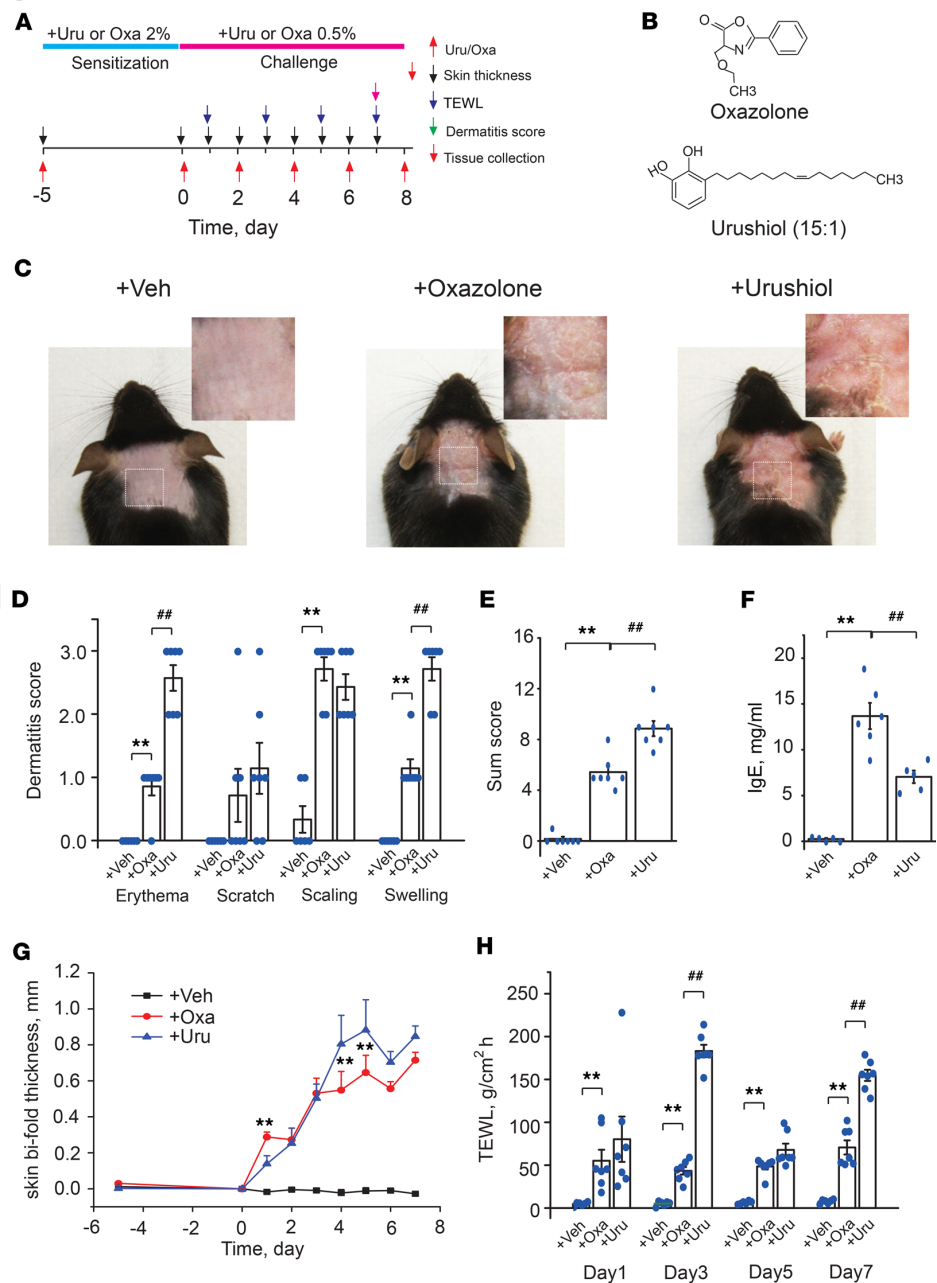
Establishment and characterization of the urushiol-induced mouse poison ivy ACD model. (**A**) Schematic of protocols for establishing oxazolone-induced (Oxa) and urushiol-induced (Uru) ACD in mice. Control group received vehicle (acetone) treatment only. TEWL, transepidermal water loss. (**B**) Molecular structure of oxazolone and urushiol (15:1). (**C**) Representative photographs of neck skin from mice treated with vehicle, oxazolone, or urushiol. (**D**) Dermatitis subscores of mice treated with vehicle (Veh), Oxa, or Uru. (**E**) Total dermatitis scores, derived by summation of subscores derived from **D**. (**F**) Plasma IgE level determined by ELISA. (**G**) Changes of neck skin bifold thickness. (**H**) TEWL measured at the neck skin. Data in bar graphs are expressed as mean ± SEM. *n* = 6–7 mice/group. **P < 0.01, ^##^*P* < 0.01. One- or 2-way ANOVA, followed by Tukey’s post hoc test, was used for statistical analysis.

Mouse models of oxazolone- and urushiol-induced ACD were established for comparison of inflammatory and pruritic responses (Figure 1, A and B). After the fifth allergen challenge, mice of both groups showed typical hallmarks of ACD, including skin erythema, skin scratches, scaling, and edema (Figure 1, C–E). Both groups showed significantly upregulated serum IgE (Figure 1F). Bifold skin thickness and TEWL were also significantly increased in both groups over the challenge period (Figure 1, G and H). Despite similar dermatological manifestations between both groups, some differences were noted. First, the urushiol group displayed much stronger erythema and skin edema reactions than the oxazolone group, resulting in higher summarized dermatitis score (Figure 1, C–E and G). Second, the urushiol group had much higher TEWL than the oxazolone group at certain time points during the challenge period but lower serum IgE (Figure 1, F and H).

Mice from both groups gradually developed scratching behaviors toward the nape of neck (Supplemental Figure 1A; supplemental material available online with this article; https://doi.org/10.1172/jci.insight.124497DS1). Urushiol- and oxazolone-challenged mice both responded with long-lasting scratching behavior for up to 24 hours (Supplemental Figure 1B). To further distinguish itch from pain behaviors caused by urushiol-induced ACD, we used the mouse cheek ACD model, which distinguishes pain (forepaw wiping) and itch (hind paw scratching) behavior (19). After the fifth challenge, urushiol-challenged mice developed both significant scratching and wiping behaviors compared with control mice (Supplemental Figure 1, C and D). These results suggest that urushiol-induced ACD causes mixed itch and pain sensations, which mimics the sensations of poison ivy–affected human patients (8, 20). Oxazolone-challenged mice developed scratching and wiping behaviors as well (Supplemental Figure 1, C and D).

Next, we compared the histological changes of the inflamed skin in both models. We observed characteristic epidermal thickening, spongiosis, and eosinophilia, which are typical features of human ACD, in both oxazolone and urushiol groups (Supplemental Figure 2, A and B). The oxazolone group showed significantly more spongiosis whereas the urushiol group showed significantly more eosinophilia compared with each other (Supplemental Figure 2, A and B). No significant difference was observed between oxazolone and urushiol groups in epidermal thickness (Supplemental Figure 2, A and B). Toluidine staining revealed that the number of mast cells was significantly increased in both groups, with no significant difference between the groups. But the oxazolone group showed a higher degranulation rate than the urushiol group (Supplemental Figure 2, A and B). Immunohistochemistry showed that the number of CD3^+^ T cells was increased in both groups, with no significant difference between the groups (Supplemental Figure 2, A and B).

### Skin transcriptome profiling identifies common and distinct patterns of molecular responses in oxazolone- and urushiol-induced ACD mice.

**Figure 2:**
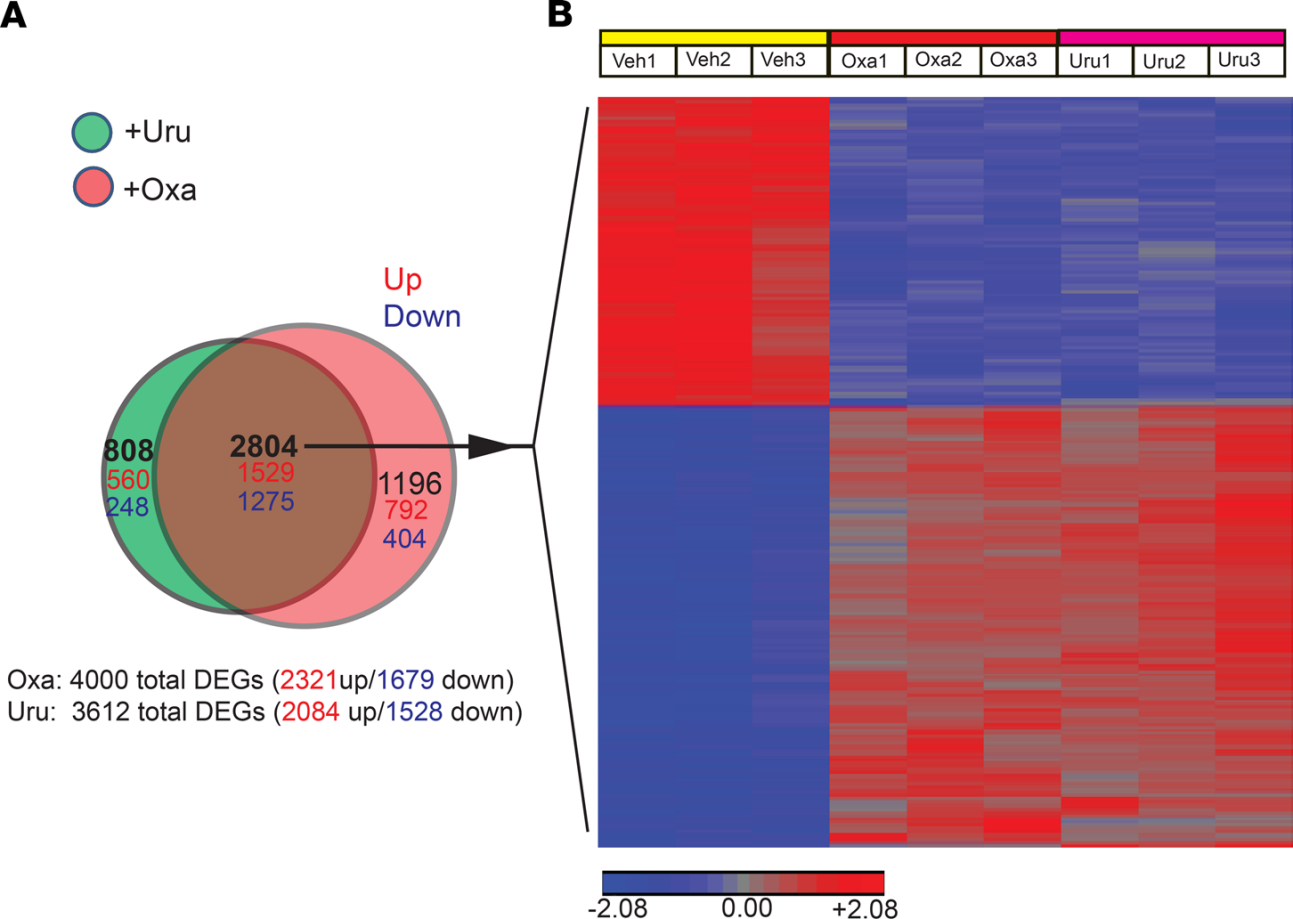
Whole-transcriptome microarray analysis of skin from oxazolone- and urushiol-induced ACD mouse models. (**A**) Venn diagram showing the overlapping of DEGs between oxazolone- and urushiol-treated groups (red, upregulated; blue, downregulated). (**B**) Heatmap showing the common DEG (2804 genes) set shared by oxazolone- and urushiol-treated groups. *n* = 3 mice/group.

To gain a comprehensive understanding of the molecular responses to oxazolone and urushiol, we examined the gene expression profiles of mouse skin by transcriptome microarray analysis. We successfully obtained high-quality RNA from mouse skin (Supplemental Figure 3) that was subjected to whole-transcriptome microarray analysis, with the aim to identify differentially expressed genes (DEGs) in oxazolone- and urushiol-challenged mice compared with vehicle-treated mice (with criteria of fold change > 2 or < –2 and *P* < 0.05). Subsets of these transcriptome data were used in our recent publication (17). Here, we continued to further analyze the whole-transcriptome data in more detail. The scatter plot identified a large number of DEGs in both oxazolone and urushiol groups (Supplemental Figure 4). A total of 4000 DEGs (2321 upregulated, 1679 downregulated) in the oxazolone group and 3612 DEGs in the urushiol group (2084 upregulated, 1528 downregulated) were identified. Among these DEGs, we identified a core set of 2804 (1529 upregulated, 1275 downregulated) common DEGs between the oxazolone and urushiol groups (Figure 2A). This common core gene set is displayed in the heatmap in Figure 2B. The common core gene set consists of genes that include barrier-related genes (*Krt16*, *Krt6a*, *Krt17*), immune regulators (*Cd274*), antimicrobial protein genes (*S100a8*, *S100a9*), cytokines (*Il24*, *Il36a*, *Il36g*, *Il19*, *Il33*), chemokines (*Cxcl2*, *Ccl3*), and some general inflammatory markers (*Mmp9*, *Mmp13*, *Il1b*). In addition to the common core set of DEGs, oxazolone and urushiol groups also showed distinct patterns of transcriptional changes. The oxazolone group contained 1196 (792 upregulated, 404 downregulated) distinct DEGs, and the urushiol group contained 808 (560 upregulated, 248 downregulated) distinct DEGs (Figure 2A).

Enrichment analysis using the Kyoto Encyclopedia of Genes and Genomes pathways was performed to further compare the oxazolone and urushiol models. Supplemental Figure 5 illustrates the selected pathways significantly enriched in the 2 models. The most significantly enriched pathways in both models included cytokine–cytokine receptor interactions, chemokine signaling pathways, NF-κB pathway, rheumatoid arthritis signaling, and phagosome and TNF signaling pathway. The oxazolone model showed distinct enrichments in the hematopoietic cell lineage, osteoclast differentiation, Toll-like receptor signaling pathway, and others, whereas the urushiol model displayed distinct enrichments in some amino acid and lipid metabolic and p53 signaling pathways, among others.

### Distinct immune mediator gene transcription profiles in the skin of oxazolone and urushiol ACD models.

**Figure 3:**
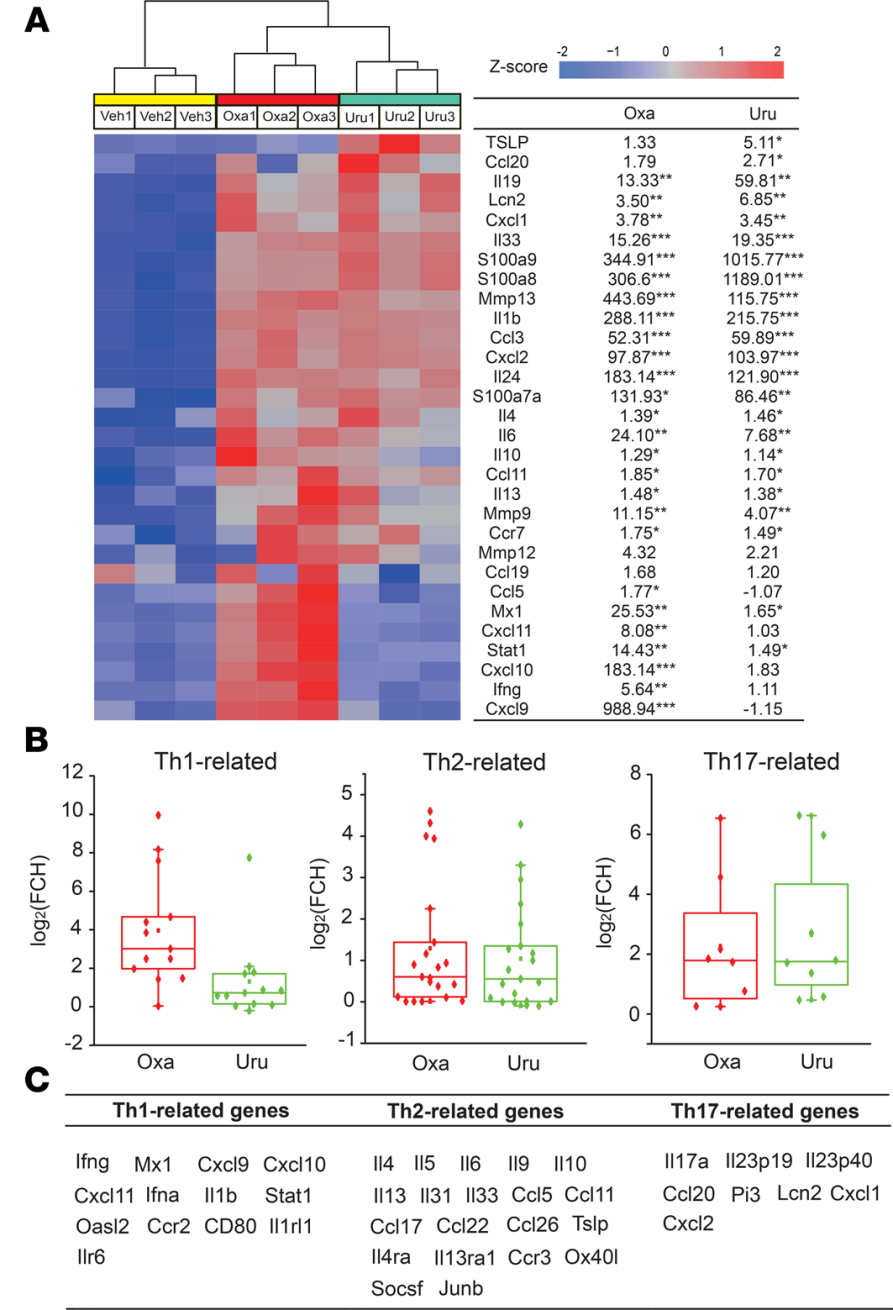
Common and unique DEGs in skin of oxazolone- and urushiol-treated groups compared with the vehicle group, identified by transcriptome microarray. (**A**) Heatmap showing the expression of immune-related genes in mouse skin. Samples in the heatmap are grouped by treatments. (**B**) Box plots of representative gene expression changes across major immune pathways represented in transcriptome microarray. The box plots depict the minimum and maximum values (whiskers), the upper and lower quartiles, and the median. The length of the box represents the interquartile range. *n* = 3 mice/group. *P < 0.05, **P < 0.01, and ***P < 0.001 vs. control (acetone-treated) group. (**C**) The list of Th1, Th2 and Th17-related genes included in **B**.”

To further evaluate potential differences in immune activation in oxazolone and urushiol groups, we compared the transcription of some well-established inflammation-related genes. We found that oxazolone activated both genes involved in Th1 responses (*Ifng*, *Cxcl9*, *Cxcl10*, *Cxcl11*, *Mx1*) and genes activated during Th2 responses (*Il4*, *Il10*, *Il13*, *Il33*; Figure 3A). In contrast, urushiol mainly activated Th2-specific genes (*Il4*, *Tslp*, *Il13*, *Il33*), with Th1 genes showing minimal responses (Figure 3A). We further extended our transcriptional analysis of the major inflammatory immune pathways by including additional genes involved in Th1, Th2, and Th17 responses (Figure 3, A–C; refs. 15, 21, 22). This analysis further showed that oxazolone and urushiol activate distinct response patterns. Although oxazolone activated Th1-, Th2- and Th17-related genes, the urushiol-activated genes remained within the Th2- and Th17-related groups of genes (Figure 3, A–C). These findings imply that the immune response in ACD is allergen dependent and should not be considered a uniform immune phenomenon.

### Validation of transcriptional profiles by qPCR confirms the distinct immune activation by oxazolone and urushiol.

**Figure 4:**
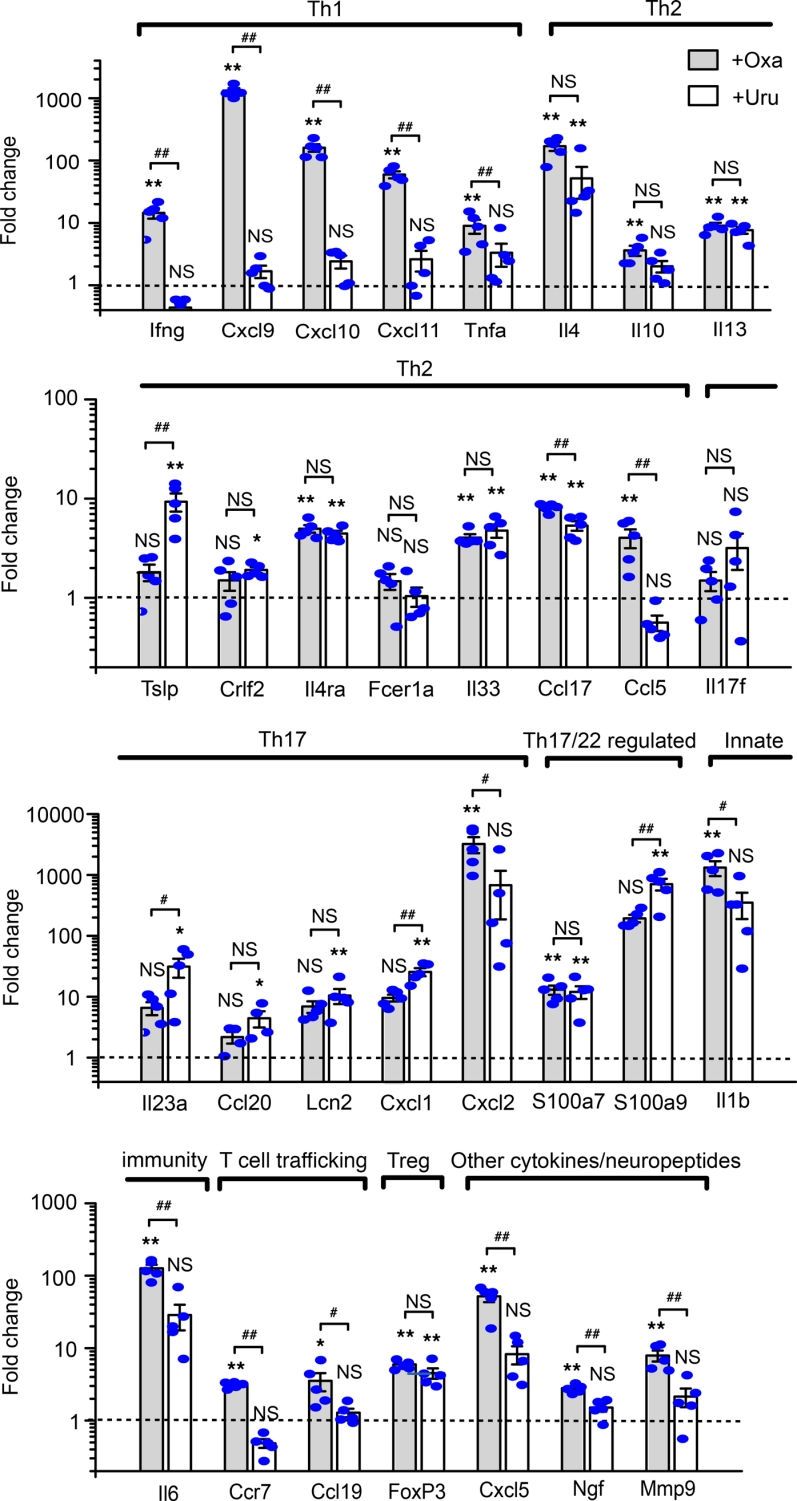
Validation of differential skin expression profiles of major cytokines and inflammatory marker genes in the oxazolone- and urushiol-induced ACD models. Real-time TaqMan qPCR data were arranged according to the major inflammatory and regulatory pathways. Gene transcripts in skin samples of oxazolone-induced (gray column) and urushiol-induced (white column) ACD mice were compared to levels in respective vehicle control mice (not shown), as determined by TaqMan real-time qPCR. β-Actin transcript levels were used as an endogenous control. Data in bar graphs are expressed as mean ± SEM. *n* = 5 mice/group. *P < 0.05, and **P < 0.01 vs. vehicle control group; ^#^*P* < 0.05, and ^##^*P* < 0.01 vs. urushiol group. NS, no significance. One-way ANOVA followed by Tukey’s post hoc test was used for statistical analysis.

TaqMan qPCR of skin cDNA was used to validate transcriptional regulation of representative inflammatory and immune-related genes (Figure 4). In accordance with the transcriptome microarray data, Th2-related genes (*Il13*, *Il33*, *Ccl17*), a Th17/22–related gene (*S100a7*), and a Treg-related gene (*FoxP3*) were all significantly upregulated in both models (Figure 4). The oxazolone group showed strong upregulation of Th1-related genes (*Ifng*, *Cxcl9*, *Cxcl10*, *Cxcl11*, *Tnfa*) and Th2-related genes (*Il4*, *Il10*, *IL13*, *Il33*, *Ccl17*, *Ccl5*) (Figure 4). In addition, T cell–trafficking genes (*Ccr7*, *Ccl19*), a Th17/22–related gene (*S100a7*), some other inflammatory genes (*Il1b*, *Il6*, *Cxcl5*, *Ngf*, *Mmp9*), and 1 Th17-related gene (*Cxcl2*) were increased in the oxazolone group (Figure 4). In comparison, the urushiol group showed no significant upregulation of Th1-related genes but significant upregulation of Th2-related genes (*Il4*, *Il13*, *Tslp*, *Il33*, *Ccl17*). In addition, some Th17-related genes (*Il23a*, *Ccl20*, *Lcn2*, *Cxcl1*) and Th17/22–related genes (*S100a9*) were significantly increased in the urushiol group (Figure 4). In summary, qPCR analysis largely validated the major findings from transcriptome microarray results, confirming that the oxazolone model predominantly produced a mixed Th1 and Th2 immune response, and urushiol triggered a predominant Th2 response, whereas both groups produced a slight Th17 response.

### Distinct patterns of endogenous itch mediators in the oxazolone- and urushiol-induced ACD models.

**Figure 5:**
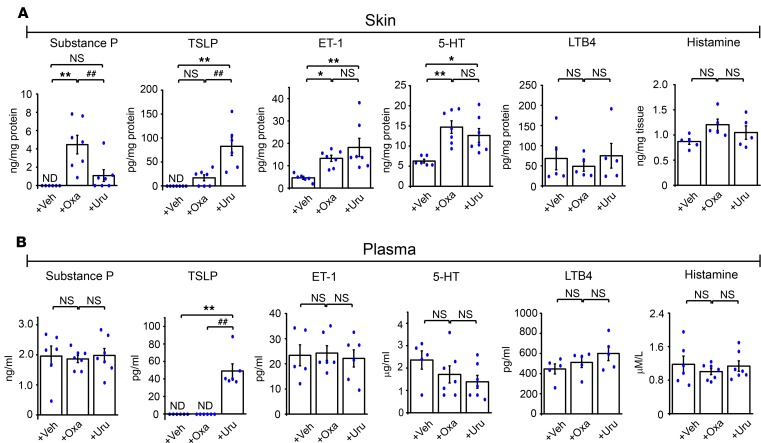
Levels of major endogenous itch mediators in the skin and plasma of oxazolone and urushiol ACD models, measured by ELISA. (**A**) Average protein concentrations of substance P, TSLP, 5-HT, ET-1, LTB4, and histamine in neck skin extracts of oxazolone-induced and urushiol-induced ACD mice compared with vehicle control mice. (**B**) The concentrations of itch mediators in the plasma of the same group of mice. Data in bar graphs are expressed as mean ± SEM. *n* = 5–7 mice/group. *P < 0.05, **P < 0.01, and ^##^*P* < 0.01. NS, no significance. One-way ANOVA followed by Tukey’s post hoc test was used for statistical analysis.

In our microarray and qPCR analysis, we observed that the *Tslp* gene was specifically upregulated in the inflamed skin of the urushiol model but not in the oxazolone model. Thymic stromal lymphopoietin (TSLP) was identified as an endogenous itch mediator that causes itch responses by activating TSLP receptors expressed in pruriceptors, the peripheral sensory neurons that detect pruritogens and initiate the sensation of itch (23). Based on our observation of differential TSLP expression, we hypothesized that oxazolone and urushiol may induce distinct expression and release patterns of endogenous itch mediators. To test this hypothesis, we used ELISA to measure levels of major well-established endogenous itch mediators, including the peptides substance P, TSLP, and endothelin (ET-1); the transmitters serotonin (5-HT) and histamine; and the lipid mediator leukotriene B4 (LTB4), in the inflamed skin and plasma of mice from both models. Substance P, ET-1, and 5-HT, but not TSLP, LTB4, or histamine, were significantly increased in the inflamed skin of the oxazolone group (Figure 5A). In contrast, TSLP, ET-1, and 5-HT, but not substance P, LTB4, or histamine, were significantly increased in the inflamed skin of the urushiol group (Figure 5A). None of the abovementioned endogenous itch mediators was increased in the plasma of the oxazolone group, whereas TSLP was significantly increased in the plasma of the urushiol model (Figure 5B). These results identified substance P and TSLP as key, distinct endogenous itch mediators released in the inflamed skin or plasma of the oxazolone and urushiol models, respectively, implying that, in addition to the distinct immune responses, distinct pruritic mechanisms may be activated in these 2 models.

### Pruritogenic signaling pathways contributing to scratching behavior in the urushiol-induced ACD model.

**Figure 6:**
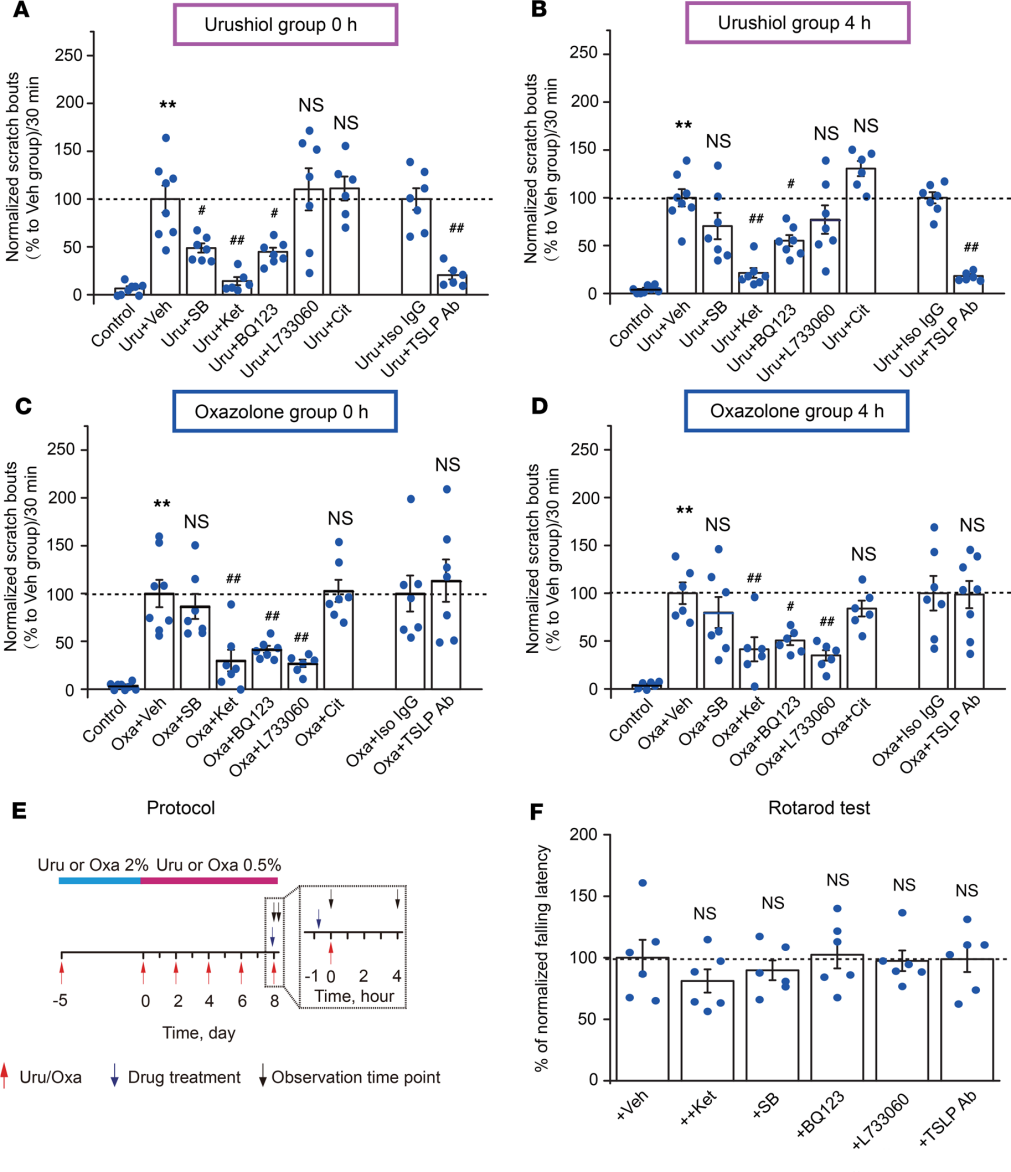
Pharmacological targeting of pruritogenic pathways in urushiol and oxazolone ACD models. (**A**) Comparison of scratching behavior in mice after injection of 5-HT_7_ receptor antagonist SB269970 (SB), 5-HT_2A_ receptor antagonist ketanserin (Ket), ET_A_ receptor antagonist BQ123, H_1_ receptor antagonist cetirizine, or TSLP-neutralizing antibody (TSLP Ab) during the first 30 minutes after urushiol challenge. (**B**) Scratching behavior of the same group of mice as in **A**, analyzed 4 hours after urushiol challenge. (**C**) Comparison of scratching behavior in mice after injection of above pharmacological reagents during the first 30 minutes after oxazolone challenge. (**D**) Scratching behavior of the same group of mice as in **C**, analyzed 4 hours after oxazolone challenge. Scratching bouts were normalized to those of vehicle-treated urushiol or oxazolone-challenged mice (Uru + Veh or Oxa + Veh). Control group represents behavior of mice challenged with vehicle (acetone) alone. *n* = 6–8 mice/group. **P < 0.01 vs. control; ^#^*P* < 0.05, and ^##^*P* < 0.01 vs. Uru + Veh, Uru + Iso IgG, or Oxa + Veh and Oxa + IgG groups. NS, no significance vs. Uru + Veh, Uru + Iso IgG, or Oxa + Veh and Oxa + IgG groups. (**E**) Schematic protocol for testing the effects of different pharmacological interventions on scratching behavior of urushiol or oxazolone groups of mice. The time scale of day 8 is expanded to visualize treatment and observation time points. (**F**) Locomotor functions of mice after being treated with different pharmacological reagents mentioned above. Data in bar graphs are expressed as mean ± SEM. *n* = 6 mice/group. NS, no significance versus Veh group. Student’s *t* test or 1-way ANOVA followed by Tukey’s post hoc test was used for statistical analysis.

In a previous study using the oxazolone ACD model, we used pharmacological and genetic approaches to identify key pruritic pathways contributing to itch-related behaviors (16). To explore whether the same pathways are also active in the urushiol model, mice were injected 45 minutes before the last urushiol or oxazolone challenge with 5-HT_7_ receptor antagonist SB269970 (SB, 30 mg/kg, i.p.), 5-HT_2A_ receptor antagonist ketanserin (Ket, 3 mg/kg, i.p.), ET_A_ receptor antagonist BQ123 (1 mg/kg, i.p.), NK_1_ receptor antagonist L733060 (20 mg/kg, i.p.), H_1_ receptor antagonist cetirizine (10 mg/kg, i.p.), or TSLP-neutralizing antibody (TSLP Ab, 25 μg/mouse, intradermal). SB, Ket, BQ123, and TSLP Ab strongly inhibited the scratching behavior immediately after urushiol challenge (Figure 6A). Four hours after the urushiol challenge, mice injected with Ket, BQ123, or TSLP Ab continued to show significantly reduced scratching behavior compared with vehicle- or isotype control IgG–treated mice (Figure 6B). In contrast, L733060 and cetirizine did not inhibit scratching behavior at 0 and 4 hours, suggesting that substance P NK_1_ receptors and histamine H_1_ receptor are not involved in itch signaling in the urushiol model (Figure 6, A and B).

In the oxazolone model, Ket, BQ123, and L733060 all effectively reduced the scratching behavior immediately (0 hours) and 4 hours after oxazolone challenge (Figure 6, C and D), supporting our previous findings (16). SB, TSLP Ab, and cetirizine did not inhibit scratching behavior at 0- and 4-hour time points, suggesting that the 5-HT_7_ receptor, histamine H_1_ receptor, and TSLP are not involved in the behavioral responses of the oxazolone model. Supplemental Table 2 contains the complete raw data quantifying scratching behavior used to generate Figure 6, A–D. Figure 6E illustrates the protocol used for pharmacological interventions and time points of observation. SB, Ket, BQ123, L733060, and TSLP-neutralizing Ab at dosages used above did not affect the locomotor activity of the mice tested by rotarod assay (Figure 6F). Overall, these findings demonstrate that TSLP, 5-HT, and ET-1 signaling promote itch-related scratching behavior in the urushiol-induced ACD mouse model and that both common and distinct pruritogenic mechanisms are engaged in the oxazolone and urushiol models.

## Discussion

Our study identified distinct immune responses and pruritic mechanisms in oxazolone- and urushiol-induced ACD models. Distinct immune polarizations to specific allergens have been reported both in human ACD patients and in animal ACD models (14, 15). Experimental allergens, such as DNFB and 2,4,6-trinitrochlorobenzene (TNCB), produce mainly Th1-type immune responses in rodents, whereas another experimental allergen, FITC, triggers a Th2-biased immune response (24–26). In humans, different allergens, such as nickel, latex rubber, and fragrances, can cause distinct immune pathway activation (15).

The Th1 immune response tends to produce proinflammatory responses and perpetuates autoimmune responses (27). IFN-γ is the main Th1 cytokine. The Th2-type cytokines include IL-4, IL-5, and IL-13, which are associated with the production of IgE and eosinophilic responses in atopy, and also IL-10, which promotes antiinflammatory responses (27). The Th17 immune response, which produces the major proinflammatory cytokine, IL-17, orchestrates the pathogenesis of inflammatory and autoimmune diseases (28). Here, transcriptome microarray and qPCR profiling of the skin revealed that urushiol exposures triggered a predominantly Th2-biased immune response. This is in sharp contrast with the oxazolone model, which exhibits a Th1/Th2 mixed immune response. Until now, we believe no comprehensive studies of the exact immune pathways in human poison ivy ACD patients have been published. However, early studies in human poison ivy patients observed that levels of IL-4 and IL-10, 2 typical Th2 cytokines, instead of IFN-γ, the primary Th1 cytokine, were increased in skin blisters (29, 30). More recently, an in vitro study reported that urushiol significantly increased the number of Th17 cells in cultured CD45RO^+^ memory T cells isolated from peripheral blood of poison ivy ACD patients compared with samples from healthy volunteers (31). These findings suggest that human poison ivy ACD may involve Th2- and Th17-type but not Th1-type immune responses. Although more detailed human studies are needed, these similarities imply that the mouse urushiol ACD model can replicate at least some of the key features of the immune responses of humans to poison ivy exposures. Future studies are needed to compare the biomarkers in the mouse urushiol model with those in poison ivy–affected patients.

Recent studies in animal models identified histamine-independent pruritogenic pathways that trigger itch. Clinical studies in poison ivy patients have shown that antihistamines are ineffective for treating itch (10). Our urushiol mouse ACD model replicated this finding. Urushiol challenge did not increase histamine levels in mouse skin, and itch-related behavior was insensitive to antihistamine treatment, supporting a predominant role of histamine-independent itch pathways. Recently, we identified a crucial role for neuronal IL-33/ST2 signaling in mediating itch in the urushiol ACD mouse model. Blocking IL-33/ST2 signaling in sensory neurons significantly reduced itch-related behavior in urushiol-challenged mice. However, residual itch behavior remained, suggesting that other pruritogenic pathways continue to signal in the urushiol ACD model (17, 18). In the present study, we screened for the presence of additional endogenous itch mediators in the skin and plasma of the urushiol model, identifying TSLP, 5-HT, and ET-1 as candidates potentially contributing to itch-related behavior.

TSLP, a key cytokine controlling T cell function, was found to cause itch behavior in mice by activating TSLP receptors expressed in sensory neurons (23). Here, we demonstrate that neutralizing TSLP by a monoclonal antibody significantly attenuated scratching behavior in urushiol-challenged mice, suggesting TSLP plays a key role in mediating the pruritus response of poison ivy ACD. In contrast with the urushiol model, the oxazolone model produced only minimal amounts of TSLP in skin, and none could be detected in plasma. Consistent with this finding, neutralizing TSLP did not reduce the itch behavior in the oxazolone model. Instead, control experiments in the oxazolone model validated our previous findings, showing elevated skin levels of neuropeptide substance P and robust antipruritic activity of inhibitors of NK_1_ receptors for substance P (16). Intriguingly, the urushiol model did not show significantly increased substance P in the skin, and inhibition of NK_1_ receptors failed to reduce itch behavior. These results demonstrated that the 2 models engage distinct pruritogenic pathways. Although substance P antagonism was shown to reduce chronic itch in pruritic conditions in clinical trials (32, 33), our findings suggest that efficacy may depend on the allergen and the immune response of the individual patient.

Itch-related behavior in the urushiol model also responded to pharmacological interference with serotonergic and endothelin signaling. Selective inhibitors were used to probe the involvement of 5-HT_7_ and 5-HT_2A_ receptors. Inhibition of the 5-HT_7_ receptor was moderately effective shortly after urushiol challenge but not 4 hours later. Inhibition of 5-HT_2A_ receptors proved to be more efficacious throughout the observation phase. Inhibition of ET_A_ receptors also had persistent but more moderate effects on scratching behavior. In a previous study, we observed that inhibition of 5-HT_2A_ and ET_A_ receptors also reduced itch-related behavior in the oxazolone model, indicating that these itch signaling pathways are shared by these 2 models. Here, we identified several signaling systems, activated by TSLP, 5-HT, and ET-1, as potential therapeutic targets to ameliorate itch in the urushiol ACD model. More recently, TSLP-neutralizing Ab has been tested in atopic dermatitis patients in a phase II clinical trial and showed promising effects on reducing dermatitis score and itch in combination with topical corticosteroids compared with placebo combined with topical corticosteroids (34). IL-33 signaling, identified in our previous study, represents another important target (17). However, other unidentified itch mediators and mechanisms may also be involved in the itch mechanisms of the urushiol model. Recently, IL-4 has been shown to mediate itch by acting on its receptor, IL-4Rα, on sensory neurons, and deleting neuronal IL-4Rα effectively attenuates itch in a mouse model of atopic dermatitis (35). Interestingly, we found that IL-4 mRNA levels were significantly increased in the inflamed skin of the urushiol and oxazolone mouse models, suggesting IL-4 may be another target involved in itch response of these 2 models. Therefore, a diversity of itch mediators and signaling may be engaged in the overall itch behavior of the urushiol ACD model, reflecting the complexity of the itch mechanisms that are involved.

Acute scratching responses within an hour after allergen application model the itch responses immediately after exposure of a sensitized patient to the allergen, when pruritus is strongest, which has the most adverse effect on quality of life and may cause scratch-induced complications, such as wounding and subsequent cutaneous infections. We believe that pharmacological intervention in this phase will be most efficacious to prevent peak discomfort and scratching-related adverse effects, especially in children. Therefore, in the present study, we examined the effects of different pharmacological interventions on the scratching behaviors at 0- and 4-hour time points after the allergen challenge. Further work with a longer observation time frame needs be performed to explore the overall therapeutic effects of these pharmacological interventions on the scratching behavior in the urushiol mouse model.

The identity of the itch mediators in the skin of poison ivy patients still needs to be validated. TSLP and substance P may serve as unique markers to differentiate allergen-dependent immune responses in ACD and to direct therapeutic strategies. Both anti-TSLP and anti–IL-33 therapies were clinically proven to reduce asthma exacerbations and allergic dermatitis symptoms, respectively, and are moving forward in clinical development (36, 37). These treatments may represent alternative interventions for poison ivy–exposed patients presenting with exacerbations or patients resistant or intolerant to steroids.

In all, our results indicate that the urushiol model shows distinct properties from the oxazolone model, such as skin pathology, skin immunology, and pruritus mechanisms. The urushiol model recapitulates many key clinical features of human poison ivy patients, such as pruritus/pain sensation, histamine-independent itch, and skin immunology. Our study further suggests that inhibiting signaling by TSLP and other cytokines may represent alternatives to the standard steroid/antihistamine regimen for steroid-resistant or -intolerant patients and in exaggerated systemic responses to poison ivy.

## Methods

### Animals.

Experimental procedures were approved by the Institutional Animal Care and Use Committee of Duke University. Male C57BL/6 mice (6 to 8 weeks old) were purchased from Jackson Laboratory. Mice were housed at facilities accredited by the Association for Assessment and Accreditation of Laboratory Animal Care in standard environmental conditions (12-hour light/12-hour dark cycle and 23°C). Food and water were provided ad libitum.

### Urushiol- and oxazolone-induced ACD model.

Mice were sensitized by applying 2.0% urushiol (15:1, 81080, Phytolab) or oxazolone (VWR/Alfa Aesar, L00194, 30 μL, dissolved in a 4:1 mixture of acetone/olive oil (Sigma-Aldrich), to the shaved abdomen under ketamine/xylazine anesthesia. After 5 days (day 0), mice were challenged on the shaved nape of neck by painting with 0.5% urushiol or oxazolone dissolved in acetone (40 μL). On days 2, 4, 6, and 8, mice were challenged with urushiol or oxazolone, in the same way as on day 0, for a total of 5 challenges.

### Behavioral analysis.

Mice were placed in the observation chamber to acclimate for 40 minutes. Then mice were videotaped at time points of 0 and 4 hours after allergen challenge. A series of 1 or more scratching movements by the hind paw directed toward the neck area was defined as a scratching bout, which ended when the mouse either licked its hind paw or placed its hind paw back on the floor. The total number of scratching bouts was counted for 30 minutes or 1 hour. In cases where the mouse cheek model was used, the right cheek of each mouse was be shaved beforehand, and the scratching using the hind paw and wiping using the forepaw toward the cheek area was counted for 30 minutes. All behavioral tests were performed by an experimenter blinded to experimental conditions.

### Bifold skin thickness, dermatitis evaluation, and TEWL measurement.

The increase in bifold skin thickness was measured using a digital spring-loaded thickness gauge (Mitutoyo Quick Mini, 700-118-20, Mitutoyo Corp.) and was calculated by subtracting the values before sensitization from those obtained from specific days afterward. Six determinations were made at different dorsal skin sites per mouse and averaged. The severity of dermatitis was scored following criteria described previously (16): individual scores (0, none; 1, mild; 2, moderate; 3, severe) of erythema, scratch, scaling, and swelling were summed up as the dermatitis score by an experimenter blinded to treatment groups. TEWL was measured using a VapoMeter (Delfin Technologies) under brief anesthesia with sevoflurane.

### Skin protein isolation and ELISA.

At 4 hours after the last oxazolone or urushiol challenge, mice were euthanized, and 4-mm biopsies were excised from the nape of the neck and immediately frozen in liquid nitrogen. Tissue was homogenized using a Bullet Blender (NextAdvance) in 50 mM Tris base (pH 7.4) and 150 mM NaCl with protease inhibitor and 0.2% Triton X-100. Homogenization was carried out for 20 minutes at full speed. Then samples were centrifuged at 10,000 *g* for 10 minutes at 4°C. The supernatant was used for ELISA testing of substance P (R&D Systems), ET-1 (R&D Systems), and TSLP (R&D Systems). For ELISA testing of histamine (Cayman Chemical), LTB4 (R&D Systems), and 5-HT (Beckman Coulter), the skin samples were processed according to each manufacturer’s instructions.

### RNA extraction, mouse transcriptome microarray, and data analysis.

Four hours after the last challenge, mice were sacrificed and neck skins were collected. RNA was extracted by TRIzol RNA isolating reagent (Thermo Fisher Scientific) plus RNeasy Mini Kit (Qiagen). RNA quality and purity were checked by TapeStation (Agilent) and NanoDrop (Thermo Fisher Scientific). Only RNA samples showing RNA Integrity Number greater than or equal to 8.0 and A260/230 greater than or equal to 1.5 were used for microarray. The samples were processed by Affymetrix GeneChip Mouse Transcriptome Assay 1.0. The Affymetrix Mouse Transcriptome 1.0 CEL files were imported into Affymetrix Expression Console Software, version 1.4. The CEL files were analyzed using the Gene Level–Signal Space Transformation–Robust Multi-Chip Analysis (SST RMA) normalization method (User Manual Expression Console Software 1.4, PN 702387 Rev. 4). The Gene Level–SST RMA files (CHP files) were imported into the Affymetrix Transcriptome Analysis Console 3.0 software (User Guide, Affymetrix Transcriptome Analysis Console 3.0, PN 703150, Rev. 4) for further analysis. ANOVA analysis was performed on the vehicle-, oxazolone-, and urushiol-treated groups. Any gene showing ANOVA *P* value less than 0.05 and a mean fold change more than 2 or less than –2 was considered statistically significant and included as a DEG, according to similar criteria reported before (38, 39). Data were analyzed with guidance from bioinformaticians of the Duke University Core Facility. The microarray data set has been deposited into the National Center for Biotechnology Information’s Gene Expression Omnibus repository with accession number GSE131963.

### Real-time PCR.

cDNA synthesis was performed with the high-capacity RNA-to-cDNA Kit (Applied Biosystems). Real-time PCR (qPCR) was performed with the LightCycler 480 real-time PCR system (Roche). Each sample was run in triplicate. Samples were normalized by mouse Actb. CT values were determined using LightCycler 480 software (Roche) and averaged. Relative quantification was determined by the ΔΔCT method as described previously (40, 41). TaqMan probes (Thermo Fisher Scientific) were used for all qPCR studies, and the catalog numbers are listed in Supplemental Table 1.

### Immunohistochemistry, immunofluorescent staining, and analysis.

Circular, 4-mm punch biopsies were excised from the nape of the neck and fixed in 4% formaldehyde and embedded in paraffin. Sections were cut at 4 μm, mounted onto slides, and stained with hematoxylin and eosin or toluidine, following standard protocols. For immunofluorescent staining, sections were deparaffinized and antigen was retrieved before staining. Anti-CD3 Ab was purchased from eBioscience and corresponding secondary Ab from Invitrogen (Thermo Fisher Scientific). Images were obtained by Zeiss Imager Z1 microscope and analyzed by Zen software (Zeiss). For quantification, 3 images were randomly selected per mouse tissue and averaged.

### Drug administration.

BQ123, SB, Ket, cetirizine, and L733060 were from Tocris. Mouse monoclonal TSLP-neutralizing Ab (MAB555) was from R&D Systems. The antagonists and Ab dosages were based on previous studies (16, 42, 43). Antagonists were prepared in stock solution, diluted in PBS, and injected in 5 mL/kg volume (i.p.) 45 minutes before the test. TSLP-neutralizing Ab or isotype control IgG (rat IgG, Sigma-Aldrich) was administered at 7 sites in the inflamed skin (15 μL/site) by intradermal injection via Hamilton syringe under sevoflurane anesthesia.

### Statistics.

Statistical analyses were performed between groups using Student’s *t* test, 1- or 2-way ANOVA, followed by Tukey’s post hoc test. *P* value less than 0.05 was considered significant. Data in bar graphs are expressed as mean ± SEM.

### Study approval.

All experimental procedures were approved by the Institutional Animal Care and Use Committee of Duke University, Durham, North Carolina, USA.

## Author contributions

Boyi Liu and SEJ designed and supervised this study. Boyi Liu, YT, Boyu Liu, AIC, and CY carried out the experiments, collected the data, and analyzed the data. Boyi Liu and SEJ prepared the manuscript.

## Supplementary Material

Supplemental data

Supplemental Table 2

## Version 1 06/11/2019

In-Press Preview

## Version 2 07/25/2019

Electronic publication
