# Supplementary material for: Transcriptome profiling reveals Th2 bias and identifies endogenous itch mediators in poison ivy contact dermatitis
Source: JCI Insight. 2019 Jul 25;4(14):e124497. doi: 10.1172/jci.insight.124497 (PMC6675552; doi:10.1172/jci.insight.124497)
Supplement: Supplemental data [file jciinsight-4-124497-s051.pdf]

SUPPLEMENTARY MATERIALS

Supplemental figure legends

Suppl. Fig. 1 Behavioral analysis of oxazolone and urushiol- ACD mouse models. (A) Comparison of scratching behavior towards the nape of the neck after challenge with allergen on days 1, 5 and 9. (B) Quantification of neck scratching behavior of urushiol-challenged mice during the 1st, 4th and 24th hour after the 5th challenge. (C) Hindpaw scratching bouts directed towards the cheek in the mouse urushiol ACD check model. (D) Forepaw wiping bouts directed towards the check in the urushiol cheek ACD model. n = 6-7 mice/group. $^{**}p < 0.01$ *vs*. Veh group.

Suppl. Fig. 2 Skin pathology analysis of the oxazolone and urushiol mouse ACD models. (A) Representative micrographs of neck skin sections from mice treated with vehicle, oxazolone or urushiol are shown. Skin sections were stained with hematoxylin/eosin (H&E), toluidine or anti-CD3 antibody. The inset shows magnifications from the same picture. (B) Average epidermal skin thickness, spongiosis grading, number of eosinophils, $CD3^{+}$ cells, mast cells and mast cell degranulation rate per observation field. Black arrows indicate eosinophils, mast cells or $CD3^{+}$ cells in corresponding panels. Red arrows indicate degranulated mast cells. n = 5-8 mice/group. $^{**}p < 0.01$, $^{\#}p < 0.05$ and $^{\#\#}p < 0.01$, NS: no significance.

Suppl. Fig. 3 Quality control of skin RNA used for whole transcriptome microarray analysis. Skin RNA was extracted by TRIzol plus RNeasy Mini Kit. An electropherogram of a representative skin RNA preparation is illustrated and generated on a TapeStation instrument (Agilent). 18s and 28s bands can be clearly distinguished. The calculated RIN (RNA Integrity Number) is 9.6. FU: fluorescence unit.

Suppl. Fig.4 Scatter plot comparing gene expression profiles in the skin of oxazolone and urushiol groups *vs*. vehicle-treated group. Grey dots indicate genes showing less than two-fold differences.

Suppl. Fig. 5 KEGG pathway enrichment analysis of oxazolone and urushiol ACD model. The black dotted line indicated p value of 0.05.

Suppl. Table 1 Taqman probes used in this study.

Suppl. Table 2 The raw data of scratching behaviors of pharmacological interventions of uruhsiol and oxazolone model mice.

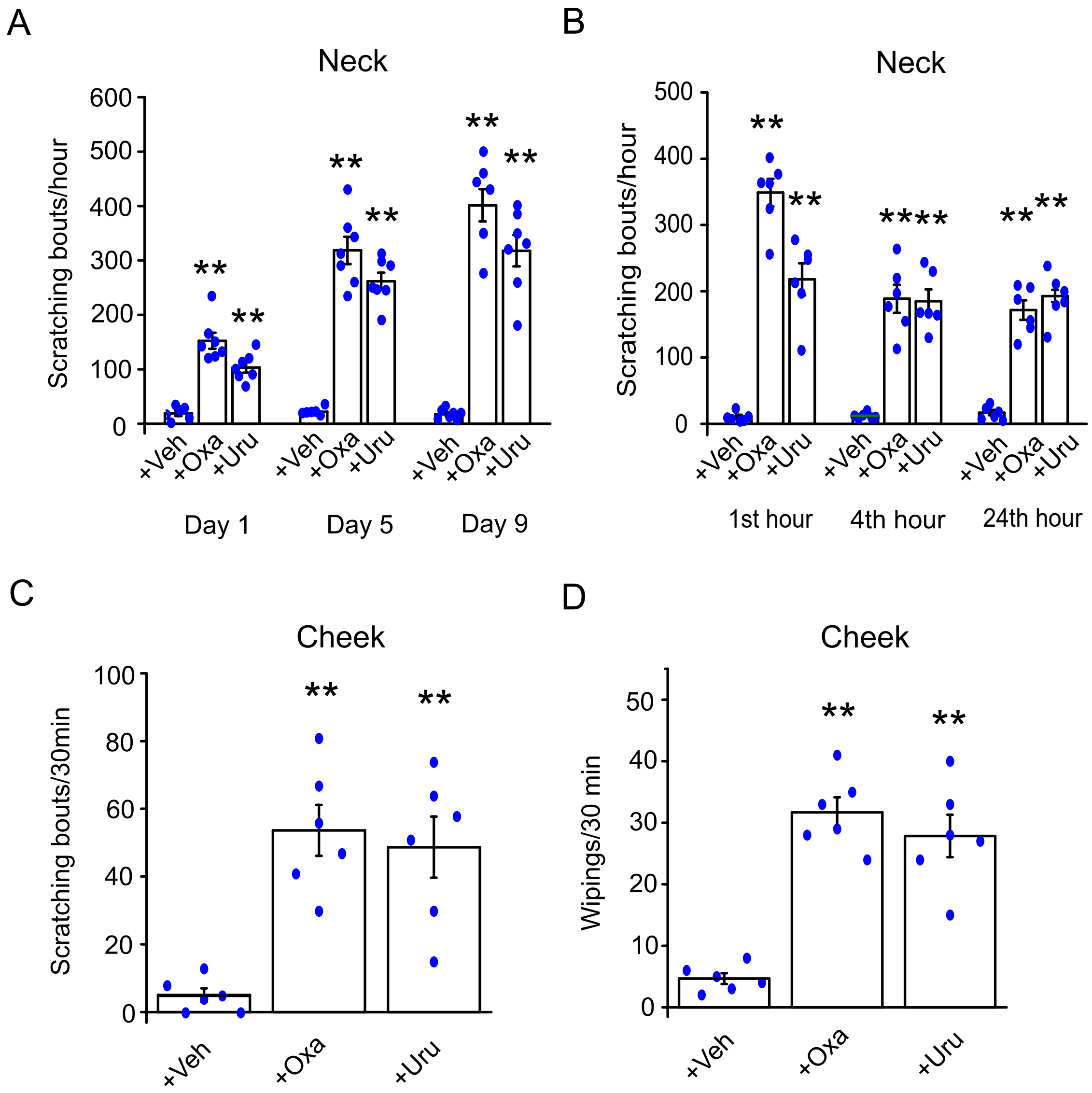

A
Neck
Scratching bouts/hour
600
500
400
300
200
100
0
**
**
**
**
**
**
+Veh
+Oxa
+Uru
Day 1
Day 5
Day 9
B
Neck
Scratching bouts/hour
500
400
300
200
100
0
**
**
****
****
1st hour
4th hour
24th hour
C
Cheek
Scratching bouts/30min
100
80
60
40
20
0
**
**
+Veh
+Oxa
+Uru
D
Cheek
Wipings/30 min
50
40
30
20
10
0
**
**
+Veh
+Oxa
+Uru

Suppl Fig. 2

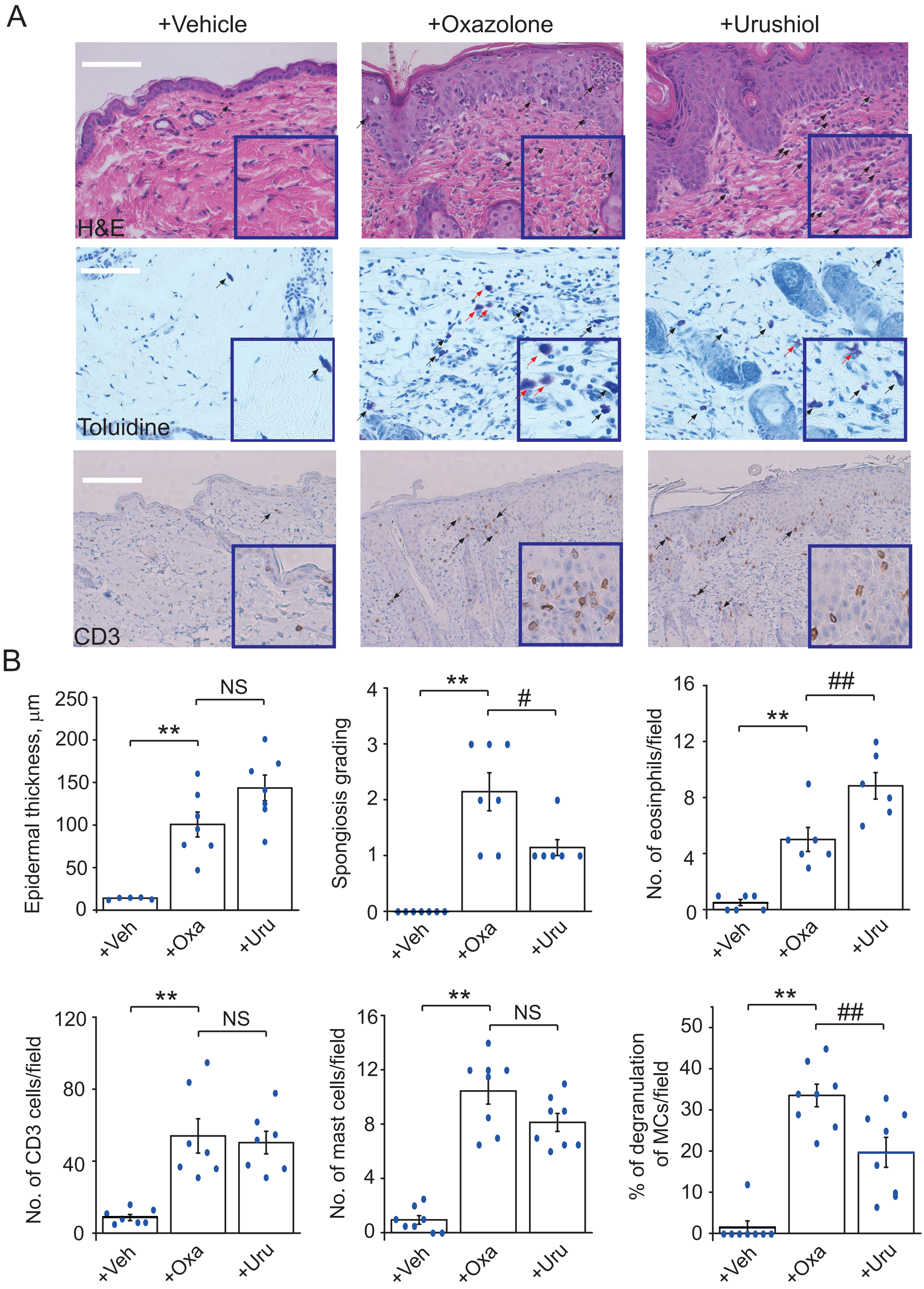

A
+Vehicle
+Oxazolone
+Urushiol
H&E
Toluidine
CD3
B
Epidermal thickness, μm
250
200
150
100
50
0
**
NS
+Veh
+Oxa
+Uru
Spongiosis grading
4
3
2
1
0
**
#
No. of eosinphils/field
16
12
8
4
0
**
##
No. of CD3 cells/field
120
80
40
0
**
NS
No. of mast cells/field
16
12
8
4
0
**
NS
% of degranulation of MCs/field
50
40
30
20
10
0
**
##

Suppl. Fig. 3

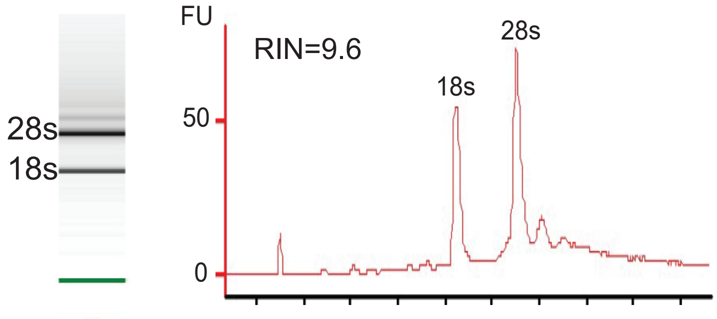

Suppl Fig. 4

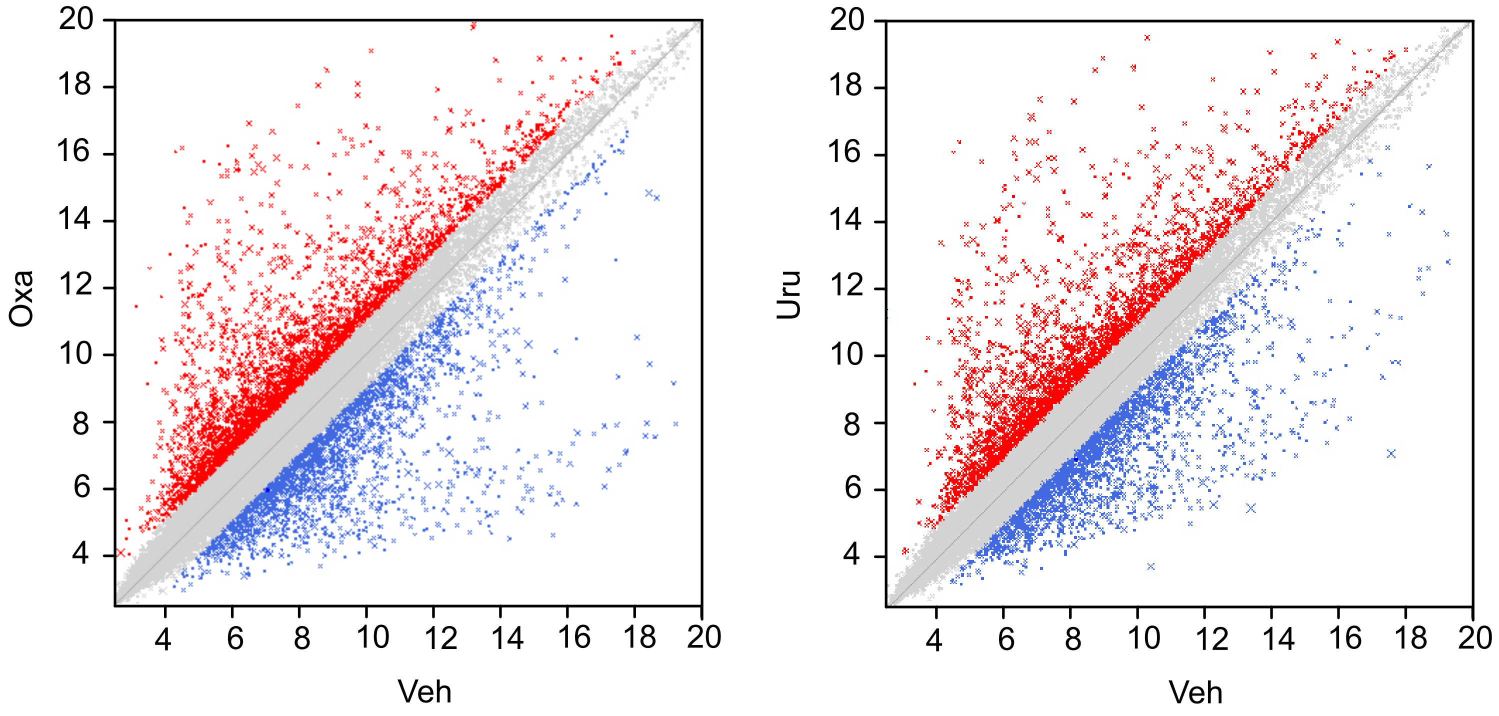

Suppl Fig. 5

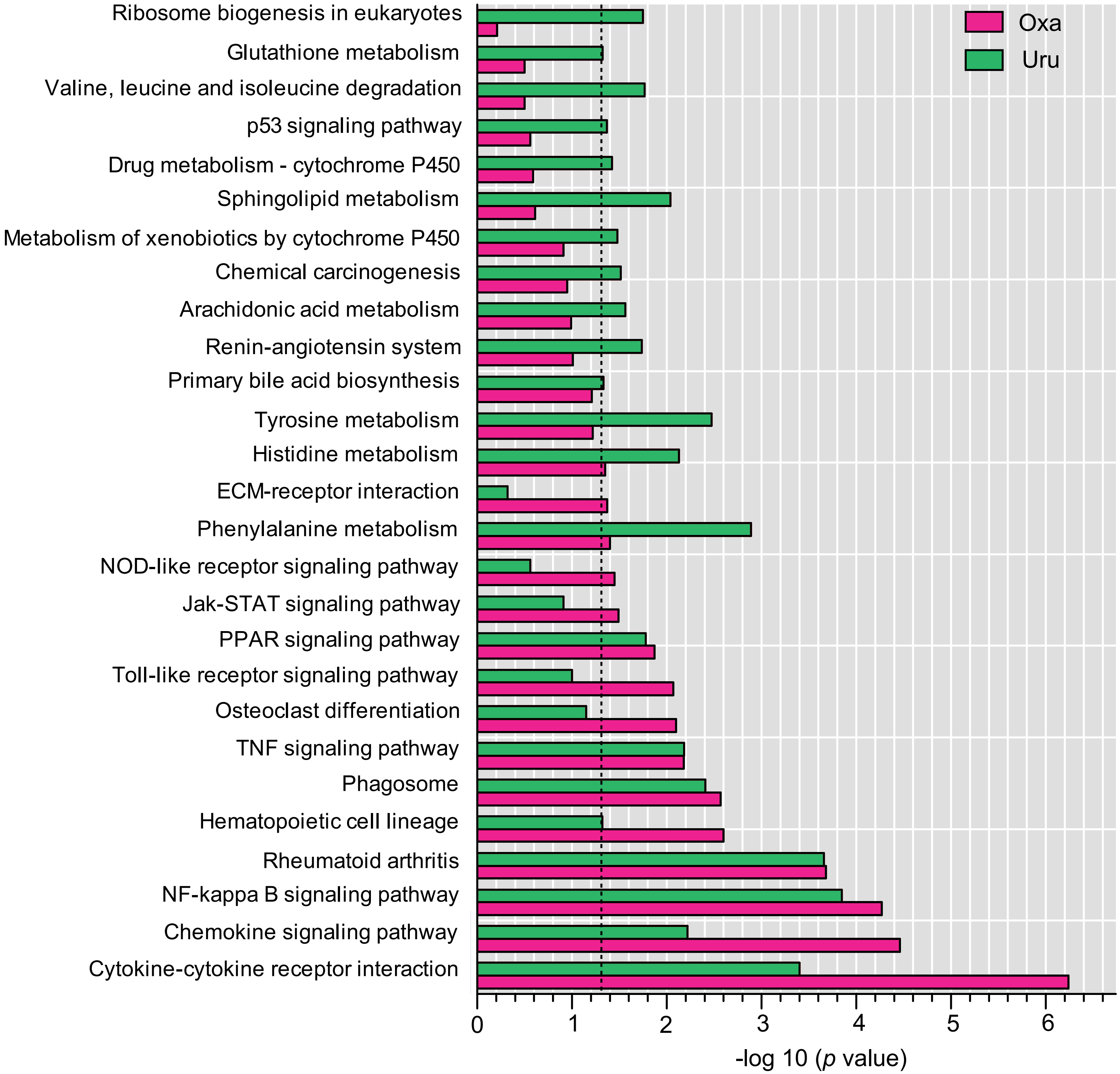
Ribosome biogenesis in eukaryotes
Glutathione metabolism
Valine, leucine and isoleucine degradation
p53 signaling pathway
Drug metabolism - cytochrome P450
Sphingolipid metabolism
Metabolism of xenobiotics by cytochrome P450
Chemical carcinogenesis
Arachidonic acid metabolism
Renin-angiotensin system
Primary bile acid biosynthesis
Tyrosine metabolism
Histidine metabolism
ECM-receptor interaction
Phenylalanine metabolism
NOD-like receptor signaling pathway
Jak-STAT signaling pathway
PPAR signaling pathway
Toll-like receptor signaling pathway
Osteoclast differentiation
TNF signaling pathway
Phagosome
Hematopoietic cell lineage
Rheumatoid arthritis
NF-kappa B signaling pathway
Chemokine signaling pathway
Cytokine-cytokine receptor interaction
Oxa
Uru
0
1
2
3
4
5
6
-log 10 (p value)

Table 1: The gene names and catalog numbers of the Taqman probes used in this study.

	Gene name	Taqman probe
1	Actb	Mm02619580_g1
2	Ifn-γ	Mm01168134_m1
3	Cxcl9	Mm00434946_m1
4	Cxcl10	Mm00445235_m1
5	Cxcl11	Mm00444662_m1
6	Tnf-α	Mm00443258_m1
7	Il-4	Mm00445259_m1
8	Il-10	Mm00439614_m1
9	Il-13	Mm00434204_m1
10	Tslp	Mm01157588_m1
11	Il-33	Mm00505403_m1
12	Ccl17	Mm01244826_m1
13	Ccl5	Mm01302427_m1
14	Il-17f	Mm00521423_m1
15	Il-23a	Mm01160011_m1
16	Ccl20	Mm01268754_m1
17	Lcn2	Mm01324470_m1
18	S100a7	Mm01218201_m1
19	S100a9	Mm00656925_m1
20	Il-1β	Mm00434228_m1
21	Il-6	Mm00446190_m1
22	Ccr7	Mm01301785_m1
23	Ccl19	Mm00839667_m1
24	Foxp3	Mm00475162_m1
25	Cxcl1	Mm04207460_m1
26	Cxcl2	Mm00436450_m1
27	Cxcl5	Mm00436451_m1
28	**Ngf**	**Mm00443039_m1**
29	**Mmp-9**	**Mm00442991_m1**
30	**Crlf2**	**Mm00497362_m1**
31	**Il4ra**	**Mm01275139_m1**
32	**Fcer1a**	**Mm00438867_m1**
